# How to Assess Risk Factors for Lead Dislodgement in Patients Receiving Cardiac Implantable Electronic Devices‐Reply‐

**DOI:** 10.1002/clc.70139

**Published:** 2025-04-30

**Authors:** Yasuhiro Matsuda, Masaharu Masuda, Hiroyuki Uematsu, Toshiaki Mano

**Affiliations:** ^1^ Kansai Rosai Hospital Cardiovascular Center Amagasaki Hyogo Japan

**Keywords:** cardiac implantable electronic device implantation, immunosuppressive therapy, lead dislodgement

The Authors' Reply:

We appreciate the comments and opinions of Dr. Kataoka and Dr. Imamura. We would like to respond to their letter.

Immunosuppressive therapy may affect lead dislodgement by suppressing adhesion between the patient's body and not only the cardiac implantable electronic device (CIED) lead tips but also the CIED lead body. As you say, focal inflammation near the site of the CIED lead tip is suppressed regardless of immunosuppressive therapy because we used steroid‐eluting leads in all patients [1]. However, CIED lead adhesions due to inflammation occur not only between the tips and the myocardium, but also between the lead body and the tricuspid valve or vessel wall [1, 2].

Regarding the incidence of lead dislodgement in patients with cardiac sarcoidosis, a previous study showed that more than half of the adverse events in implantable cardiac defibrillator implantation for cardiac sarcoidosis were lead dislodgement due to fracture [3]. In our study, two patients received immunosuppressive therapy for sarcoidosis, and 1 (50%) patient experienced lead dislodgement [1]. Additionally, cardiac sarcoidosis itself induces cardiac injury through inflammation, therefore myocardial vulnerability may also be the cause of lead dislodgement by lead tension [1, 4].

With respect to frailty, unfortunately, we did not have sufficient data on frailty in all patients. However, among the 323 (50%) patients for whom a clinical frailty score was retrospectively obtained [5], there was no significant difference in clinical frailty scores between patients with and without lead dislodgement (6 [3−7] vs. 4 [3−5] points, respectively, *p* = 0.22). In addition, there was no significant difference in clinical frailty scores between patients on regular steroids and those not taking steroids (4 [3−6] vs. 4 [3−5] points, respectively, *p* = 0.99).

As discussed in the limitations section of the manuscript, procedural strategies and implantation skills may have varied between operators in this study [1]. However, in terms of operator learning curves, years of operator experience were similar in patients with and without lead dislodgement (9 [6−11] vs. 9 [7−12] years, respectively, *p* = 0.66), as we have previously shown in the manuscript [1].

## Disclosure

Yasuhiro Matsuda has received a scholarship from the Japanese Heart Rhythm Society, Abbott and Nihon Kohden outside the submitted work.

## Ethics Statement

The protocol of this study was approved by the Kansai Rosai Hospital Institutional Review Board (Reference number: 22D104g).

## Conflicts of Interest

Yasuhiro Matsuda has received a scholarship from the Japanese Heart Rhythm Society, Abbott and Nihon Kohden outside the submitted work.

## Data Availability

The authors have nothing to report.
